# Milk Fat Globule Membrane Alleviates Short Bowel Syndrome-Associated Liver Injury in Rats Through Inhibiting Autophagy and NLRP3 Inflammasome Activation

**DOI:** 10.3389/fnut.2022.758762

**Published:** 2022-03-04

**Authors:** Zhicai Yu, Shanshan Huang, Ying Li, Yang Niu, Honghao Chen, Jiang Wu

**Affiliations:** ^1^Department of Clinical Nutrition, Huadong Hospital Affiliated to Fudan University, Shanghai, China; ^2^Shanghai Key Laboratory of Pediatric Gastroenterology and Nutrition, Shanghai, China; ^3^Department of Clinical Nutrition, Xinhua Hospital Affiliated to Shanghai Jiaotong University School of Medicine, Shanghai, China; ^4^Guangzhou Women and Children's Medical Center Affiliated to Guangzhou Medical University, Guangzhou, China

**Keywords:** short bowel syndrome-associated liver disease, endotoxin, NLRP3 inflammasome, autophagy, milk fat globule membrane

## Abstract

**Background:**

The milk fat globule membrane (MFGM), a tri-layer membrane structure surrounding the milk fat globule, has been shown to have immune-modulating properties. This study aimed to investigate the effects of MFGM supplementation in a rat model of short bowel syndrome (SBS) associated liver disease and its possible mechanisms.

**Materials and Methods:**

Twenty one male Sprague-Dawley rats were randomly divided into three groups: Sham, SBS (underwent massive small bowel resection), and SBS+MFGM (SBS rats supplemented with 1.5 g/kg/d MFGM). Liver pathology, myeloperoxidase (MPO) staining, serum levels of aspartate aminotransferase (AST)/alanine aminotransferase (ALT), endotoxin concentration, protein expression of autophagy and nucleotide binding oligomerization domain, leucine-rich repeat and pyrin domain-containing protein 3 (NLRP3) pathway in the liver tissue were measured.

**Results:**

Both SBS and SBS + MFGM groups had higher serum levels of ALT and liver endotoxin levels than the Sham group (*P* < 0.05), with no difference detected between each other. Compared with the SBS group, the SBS+MFGM group showed lower liver pathology scores of steatosis and inflammation, less MPO positive cells and reduced expressions of NLRP3, apoptosis-associated speck-like protein containing a CARD (ASC), Caspase-1, interleukin (IL)-1β(*P* < 0.05) in the liver. Additionally, the expression of Beclin-1 and microtubule-associated protein1 light chain 3(LC3) B, the fluorescence intensity of NLRP3 and LC3B in the SBS + MFGM group were lower than the SBS group (*P* < 0.05). The LC3B expression was positively correlated with the NLRP3 level.

**Conclusion:**

Enteral supplementation of MFGM help to alleviate liver injury in SBS rats, which might be related to inhibition of aberrant activation of autophagy and NLRP3 inflammasome pathways.

## Introduction

Short bowel syndrome-associated liver disease (SBS-ALD) is one of the most common complications in patients with short bowel syndrome (SBS), which might progress to severe liver injuries such as fibrosis or even end-stage liver diseases (1). The causes and pathogenesis of SBS-ALD is complex and its mechanisms still remain unclear. Previous research suggests a multifactorial pathogenesis, including long-term use of parenteral nutrition (PN) and its related intestinal villus atrophy, oxidative stress, intestinal bacteria overgrowth and translocation (2–4). Studies indicated that small bowel resection (SBR) itself was also a key contributing factor for liver injuries (5, 6). For instance, Pereira-Fantini et al. found that piglets after massive SBR developed liver pathological changes including hepatocyte ballooning, extensive fibrosis in the portal area, and bile duct hyperplasia without receiving PN support (6).

Recently, several lines of evidence have highlighted the pivotal role of leucine-rich repeat and pyrin domain-containing protein 3 (NLRP3) inflammasome in liver diseases, such as viral hepatitis and sterile inflammatory liver injury (7, 8). NLRP3 is an intracellular sensor that could be activated by a broad range of dangerous signals, such as bacterial RNA and lipopolysaccharide (LPS), resulting in the formation and activation of the NLRP3 inflammasome and the release of the pro-inflammatory cytokines IL-1β and IL-18 (9, 10). Accumulating evidence also suggests that autophagy, a cellular waste removal and rejuvenation process, has a cross-talk with the NLRP3 pathway in numerous biological and pathological processes, particularly in infection and inflammation (11, 12). Tao et al. found that macrophages with microtubule-associated protein1 light chain 3 (LC3) depletion displayed a higher level of the cleaved form of caspase-1 and IL-1β in response to LPS and ATP treatment (13). Another study showed that pretreating HSC-T6 hepatic stellate cells with 3-methyladenine (3-MA), an autophagy inhibitor, decreased the protein expression of NLRP3 and IL-1β, thereby attenuated the NaAsO_2_-induced liver damage (14). However, the changes in autophagy and the NLRP3 inflammasome pathway in the progression of liver injury in SBS setting still remains to be explored.

The milk fat globule membrane (MFGM), a protein-lipid complex membrane structure, contains various bioactive nutrients that have been shown to have protective effects in several liver injury models (15, 16). An *in-vivo* experiment found that intravenous administration of an MFGM component-milk fat globule epidermal growth factor 8 (MFG-E8), significantly attenuated liver inflammation and oxidative stress in an animal model of hepatic ischemia-reperfusion (17). Supplementation of sphingomyelin, a major lipid component of MFGM, reduced circulating LPS content and liver steatosis in mice with high-fat-diet-induced obesity (15). Our former study showed that enteral supplementation with MFGM in SBS rats inhibited the activation of NLRP3 inflammasome in ileum and colon tissues and enhanced the intestinal barrier function (18). Studies also showed that some of the MFGM ingredients might have regulating effects on autophagy activity (19, 20), for instance, reconstructing CD36 expression in CD36^−^/^−^ mice was coupled with decreased autophagy in the liver than CD36^−^/^−^ mice treated with a high-fat diet (20). Collectively, these studies showed that MFGM or some of its components might play an important role in regulating the activities of autophagy and NLRP3 inflammasome. The present study aimed to investigate the effect of enteral MFGM supplementation on liver injury in an SBS-ALD model and to explore its potential mechanisms.

## Materials and Methods

### Animals and Experimental Design

This experimental protocol was approved by the Animal Committee of Xinhua Hospital. Five-week-old male Sprague Dawley (SD) rats were purchased from the Shanghai Rodent Laboratory Animal Center. All rats were raised at a conventional condition (20-24°C and a 12 h light/dark cycle) with free access to standard chow and water. The animals were acclimatized to the environment for a week and then randomly divided into the following three groups: Sham, SBS, and SBS + MFGM (*n* = 7 per group). The operative procedure used in this experiment had been described previously (18). Briefly, both SBS and SBS+MFGM rats underwent 80% proximal SBR (removal of the small intestine from 5 cm distal to the ligament of Treitz to 10 cm proximal to the ileocecal valve), and the Sham group was performed the transection and re-anastomosis operation at the site 10 cm proximal to the ileocecal valve. Rats in the SBS+MFGM group were gavaged with 1.5 g/kg/d of MFGM (Lacprodan MFGM-10, Arla Foods Ingredients Group, Aarhus, Denmark) dissolved in 1 ml of double-distilled (dd) water, whereas those in the other two groups were received 1 ml of dd water through intragastric administration instead (18).

### Sample Collection

All rats were euthanized through intraperitoneal administration of sodium pentobarbital (100 mg/kg) at post-operation day 15 after a night of fasting and the portal blood and hepatic samples were collected. Blood samples then were centrifuged at 941 × g for 10 min to obtain the supernatants. The serum and freshly harvested liver tissues were then stored at −80°C for further analysis.

### Hepatic Histology

Fresh liver samples were rinsed with cold normal saline and fixed in 4% paraformaldehyde. Then the liver tissues were dehydrated in ethyl alcohol with gradient proportion and embedded in paraffin. After that, the liver sections of 4 μm thickness were prepared and stained with hematoxylin-eosin (HE) routinely. Hepatic histological alterations including ballooning, steatosis, and inflammation were evaluated under the light microscope (Nikon Eclipse 80i) according to an adapted quantitation method in Table 1 (21) by two pathologists who were blinded to the purposes of the study.

**Table 1:** Histopathological algorithm and scoring system (21).

	**Algorithm**	**Scoring**
Steatosis	Lipid droplets <5%	0
	Lipid droplets in 5-33%	1
	Lipid droplets in 34-66%	2
	Lipid droplets > 67%	3
Inflammation	No inflammation	0
	Inflammatory foci per 20×	1
	>2 inflammatory foci per 20×	2
Ballooning	None	0
	Few balloon cells	1
	Many balloon cells/prominent ballooning	2

### Immunohistochemistry Staining of Myeloperoxidase in the Liver Tissues

To detect the deposition of MPO in the liver, paraffin-embedded sections from liver specimens (5 μm) were dewaxed, rehydrated, and pre-treated with high pressure for antigen retrieval. Then, 3% H_2_O_2_ was used to block endogenous peroxidase activity for 20 min at room temperature. After blocking with 10% goat serum for 20 min at room temperature, the sections were incubated with primary antibody against MPO (NO. AB208670, dilution1:1000, Abcam USA, Cambridge, MA) overnight at 4°C and incubated with a goat anti-rabbit IgG HRP-linked secondary antibody (No. ab205718, dilution1:2000, Abcam USA, Cambridge, MA) for 60 min at 37°C. Subsequently, Diaminobenzidine (DAB) was used to prompt the MPO to be visualized and the hematoxylin was used to counterstaining. Then the pieces were completed by dehydration with gradient alcohol and sealed with the mounting medium. Images were captured by Nikon Eclipse 80i. MPO positive cell per field were determined as the average of MPO positive cell count in 10 random fields at 20× in each slide.

### Indicators of Liver Function

Serum levels of alanine aminotransferase (ALT) and aspartate aminotransferase (AST) were measured using the commercial kits (Jian Cheng Bioengineering Institute, Nan Jing, China). Measurements were read using a fluorescence spectrophotometer (SpectraMax, Molecular Devices, Sunnyvale, CA, USA) according to the manufacturer's instructions.

### The Liver Endotoxin Content

The endotoxin levels of the liver tissues were measured using the toxin sensor^TM^ chromogenic Limulus amebocyte lysate (LAL) endotoxin assay kit (Genscript, Nanjing, China) according to the manufacturer's instructions. All glassware, solutions, and surgical instruments used in the experiment were autoclaved at 121°C for 15 min. Around 30 mg liver tissues were homogenized in phosphate-buffered saline (PBS), then were centrifuged at 1,672 × g at 4°C for 10 min. 100 ul collected supernatant samples were used to measure the endotoxin concentration (EU/ml).

### The Protein Expressions of NLRP3-ASC-Caspase-1 Pathway in Liver

The four key proteins [NLRP3, apoptosis-associated speck-like protein containing a CARD (ASC), Cysteinyl aspartate-specific protease-1(Caspase-1), IL-1β] expression of NLRP3 inflammasome pathway in the liver tissues were measured using western blotting as previously reported (18). Liver tissues were homogenized using RIPA buffer, supplemented with phenylmethanesulfonylfluoride (100:1ratio). The protein concentration of liver tissues was measured with a BCA Protein Assay Kit (No. T9300A, Takara, Japan). Proteins were electrophoresed in sodium dodecyl sulfate-polyacrylamide (SDS-PAGE) gels and transferred onto a polyvinylidene fluoride (PVDF) membrane for incubation with corresponding primary antibodies overnight at 4°C. The primary antibodies used were anti-NLRP3 (No. ab214185, dilution1:1000, Abcam USA, Cambridge, MA), anti-ASC (No.13833, dilution1:1000, Cell Signaling Technology, Danvers, MA), anti-caspase-1(No.2225, dilution1:1000, Cell Signaling Technology, Danvers, MA), anti-IL-1β (No. ab9722, dilution1:1000, Abcam USA, Cambridge, MA) and anti-GAPDH (No.5174, dilution1:1000, Cell Signaling Technology, USA). proteins were incubated for 1 h at room temperature with a goat anti-rabbit IgG HRP-linked secondary antibody (No.7074, Cell Signaling Technology, USA) and were visualized by a Chemi Doc XRS+ system (Bio-Rad, USA) and quantified in Image Lab version 3.0 (Bio-Rad, USA).

### The Protein Expressions of Autophagy Pathway in the Liver

The three key proteins [Beclin-1, sequestosome1(SQSTM1/p62), LC3B] expression of the autophagy pathway in the liver were detected by the western blotting. The antibodies used were anti-Beclin-1(No.3738, dilution1:1000, Cell Signaling Technology, Danvers, MA), anti-P62 (No.39749, dilution1:1000, Cell Signaling Technology, Danvers, MA), anti-LC3B (No.12741, dilution1:1000, Cell Signaling Technology, Danvers, MA) and anti-GAPDH (No.5174, dilution1:1000, Cell Signaling Technology, USA). All proteins were incubated for 1 h at room temperature with a goat anti-rabbit IgG HRP-linked secondary antibody (No.7074, Cell Signaling Technology, USA) and were visualized by a Chemi Doc XRS+ system (Bio-Rad, USA) and quantified in Image Lab version 3.0 (Bio-Rad, USA).

### The Immunofluorescence Stain of NLRP3 and LC3B in the Liver

The paraffin-embedded sections were blocked by 5% BSA for 1 h and then incubated overnight at 4°C with the primary antibodies anti-NLRP3 (No. ab214185, dilution1:1000, Abcam USA, Cambridge, MA) and anti-LC3B (No.12741, dilution1:1000, Cell Signaling Technology, Danvers, MA), respectively. Subsequently, the corresponding secondary antibodies were added to the sections for 1 h at room temperature and the 4′,6-diamidino-2-phenylindole (DAPI) was used to counterstaining the cell nuclei. Neutral gum was administered to seal the sections. Images were examined under a confocal microscope (Nikon Eclipse 80i).

### Statistical Analysis

The sample size was calculated based on the results of intestinal permeability in our previous study, as the serum fluorescein isothiocyanate-dextran-40,000 Daltons (FD-40) levels of the SBS group and SBS+MFGM group. The mean value of FD-40 in the SBS group was 1.59 μg/ml and the SBS + MFGM group mean was 0.48 μg/ml. The standard deviation of the two groups were 0.47 and 0.29 μg/ml, respectively. The type I error rate was chosen to be 0.05 based on a two-sided test, and the sample size required was four per group based on the power (1-β) of 80%.

Data were presented as the means ± SD or medians (interquartile range). Group differences were determined by ANOVA or Kruskal-Wallis *H*-test, followed by Tukey or Dunn-Bonferroni *post hoc* test, and *P-*values of < 0.05 were considered statistically significant. All statistical tests were performed using SPSS 23.0 (IBM, Chicago, IL, USA).

## Results

### General Conditions

No significant differences were detected in the body weights of Sham (134.29 ± 4.00 g), SBS (137.28 ± 11.55 g), and SBS + MFGM (131.00 ± 10.18 g) groups before the operation. Both SBS and SBS+MFGM rats had continuously lower body weight than that in the Sham group after the operation. On post-operation day 15, the body weights of the Sham (245.28 ± 15.07g) group were higher than both the SBS (206.14 ± 25.98 g) and SBS + MFGM (205.00 ± 29.82 g) groups. No surgery-related deaths occurred in the three groups.

### Hepatic Histology and MPO Immunohistochemistry Staining

**Figure 1:**
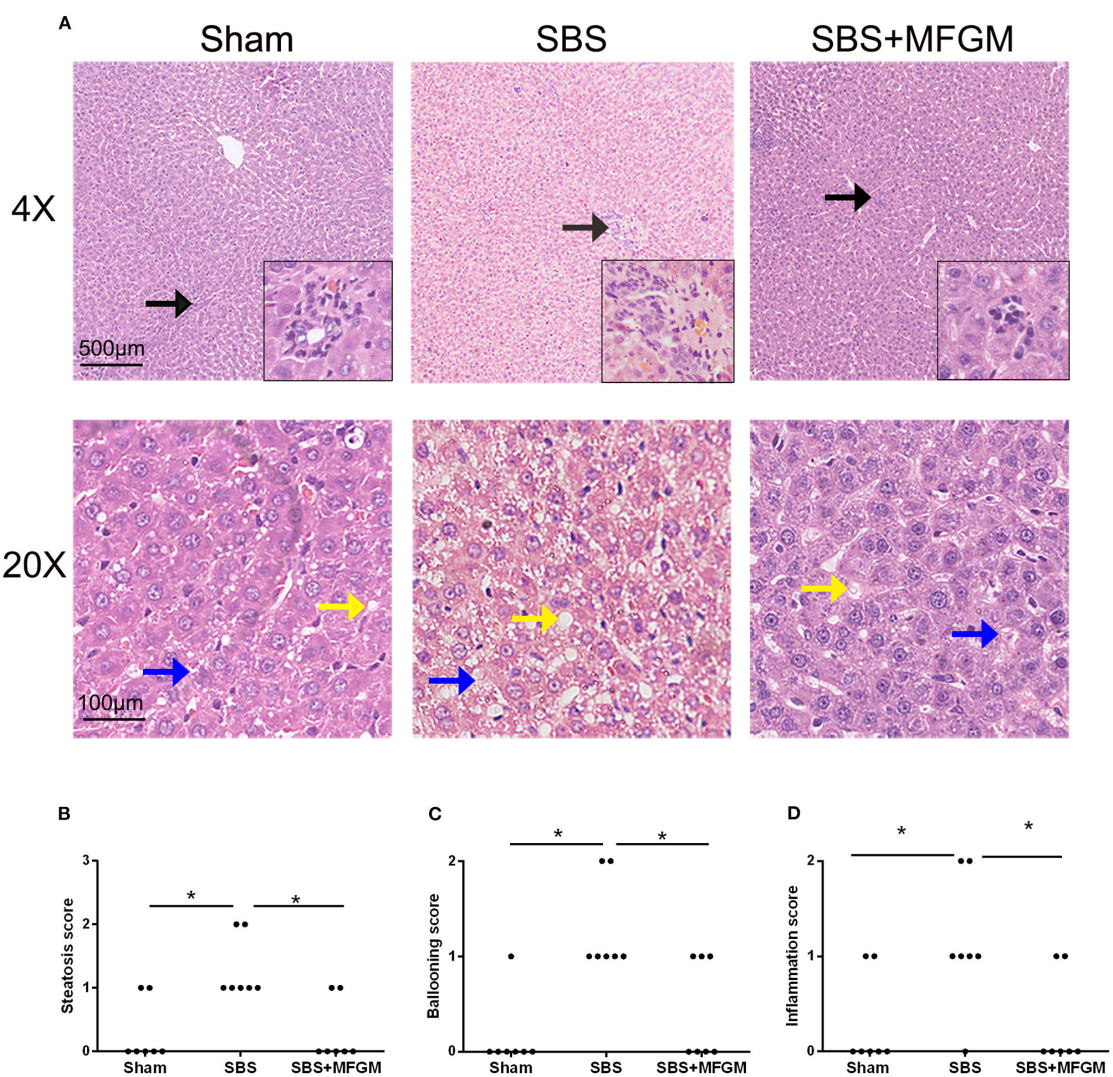
Liver images by HE-staining **(A)** and comparison of the liver histology scores in steatosis, ballooning, and inflammation **(B–D)**. The upper panels show a low-power view (×4), scale bar: 500 μm. The lower panels show a high-power view (×20), scale bar: 100 μm. Liver lesion inflammation (black arrow), steatosis (yellow arrow), and ballooning (blue arrow). Group differences were determined by Kruskal-Wallis *H*-test followed by Dunn-Bonferroni *post hoc* test and ^*^P < 0.05 indicates a difference between the two groups. MFGM, milk fat globule membrane; SBS, short bowel syndrome.

Representative histological images of the liver tissue were shown in Figure 1. Compared with the Sham group, liver histology changes in the SBS group were characterized by steatosis and hepatocyte ballooning, and inflammatory cell infiltration in the portal area. Administration of MFGM exerted protective effects on hepatic lesions in SBS rats, which was demonstrated by ameliorated levels of steatosis and inflammation (Figure 1A). Additionally, the steatosis, hepatocyte ballooning, and inflammation scores (Figures 1B–D) in the SBS + MFGM group were much lower than those in the SBS group (*P* < 0.05).

**Figure 2:**
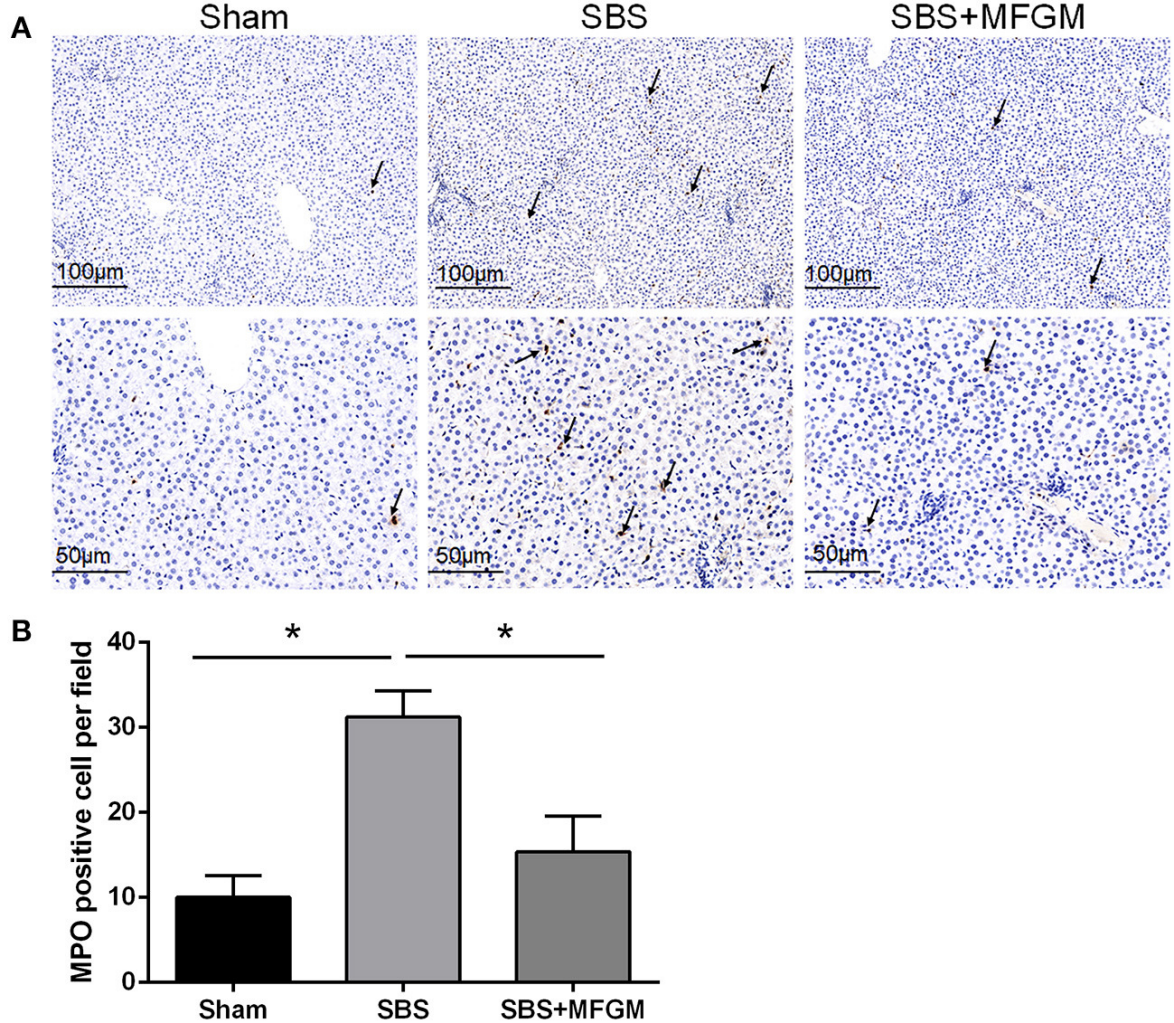
Immunohistochemistry staining of MPO in the liver **(A)**; The quantification of MPO positive cells in the liver **(B)**. Black arrows indicate MPO-positive cells. Data were presented as the means ± SD (*n* = 5-7), group differences were determined by ANOVA test followed by Tukey's *post hoc* test, and *P < 0.05 indicates a difference between the two groups. MPO, myeloperoxidase; MFGM, milk fat globule membrane; SBS, short bowel syndrome.

The immunohistochemistry staining showed more MPO positive cells in the liver tissues of the SBS rats than the other two groups (Figures 2A,B).

### Serum Levels of ALT and AST

Both the SBS (26.58 ± 3.90 U/l) and SBS + MFGM (24.30 ± 4.98 U/l) rats exhibited increased levels of ALT compared with the Sham group (15.73 ± 4.60 U/l) (*P* < 0.05), but the former two groups did not differ significantly. No difference in AST was detected among the three groups.

### The Content of Liver Endotoxin

The liver endotoxin levels in both the SBS (0.99 ± 0.12 EU/ml) and SBS + MFGM (0.89 ± 0.73 EU/ml) groups were higher than that of the Sham group (0.72 ± 0.54 EU/ml) (*P* < 0.05). The SBS + MFGM group tended to have a lower content of liver endotoxin than the SBS group (*P* = 0.083).

### The Protein Expression of the NLRP3-ASC-Caspase-1 Pathway and Autophagy Pathway in the Liver

**Figure 3:**
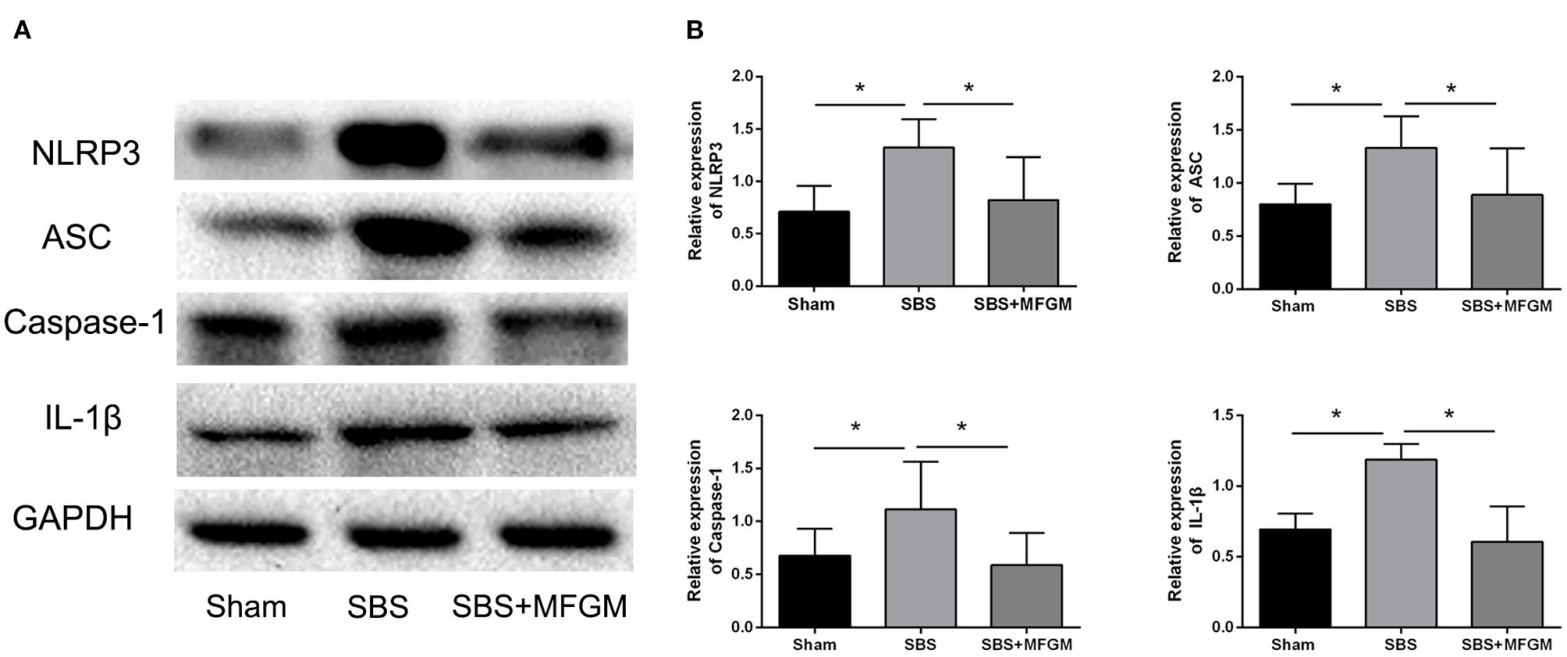
Western blotting of the NLRP3-ASC-Caspase-1 pathway proteins in the liver **(A)**; the relative expression of NLRP3 inflammasome proteins in the liver **(B)**. Data were presented as the means ± SD (*n* = 5-7), group differences were determined by ANOVA test followed by Tukey's *post hoc* test, and *P < 0.05 indicates a difference between the two groups. ASC, apoptosis-associated speck-like protein containing a CARD; Caspase-1, cysteinyl aspartate-specific protease-1; IL, interleukin; MFGM, milk fat globule membrane; NLRP3, nucleotide-binding oligomerization domain leucine-rich repeat and pyrin domain-containing protein 3; SBS, short bowel syndrome.

Compared with the Sham group, the SBS group had higher levels of NLRP3, ASC, Caspase-1, and IL-1β, which indicated the activation of the NLRP3 inflammasome (Figure 3). The SBS+MFGM group had lower levels of NLRP3, ASC, Caspase-1 and IL-1β expression, than the SBS group (Figure 3B) (*P* < 0.05). Additionally, the expression of IL-1β was positively correlated with the liver ballooning scores (Supplementary Figure S1).

**Figure 4:**
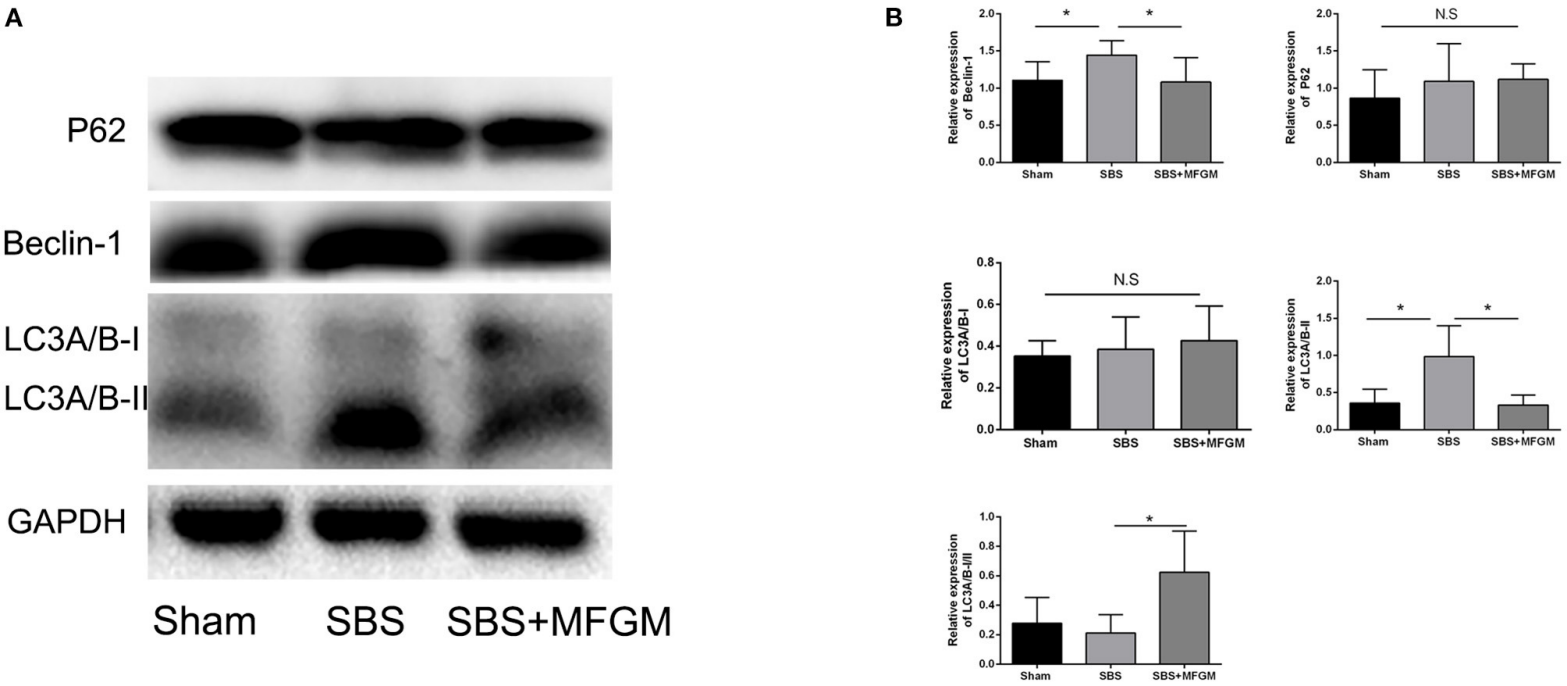
Western blotting of the autophagy pathway proteins in the liver **(A)**; relative expression of autophagy proteins in the liver **(B)**. Data were presented the means ± SD (*n* = 5-7), group differences were determined by ANOVA test followed by Tukey's *post hoc* test, and *P < 0.05 indicates a difference between the two groups, N.S. indicates no significance. LC3, microtubule-associated protein1 light chain 3; MFGM, milk fat globule membrane; SBS, short bowel syndrome.

Compared with the Sham group, SBS rats showed higher levels of Beclin-1and LC3B in the liver (*P* < 0.05), suggesting an elevated autophagy activity. SBS + MFGM group presented lowered expression of these markers than the SBS group (Figures 4A,B). Regarding the expression of P62, no statistically significant difference was found among the three groups.

**Figure 5:**
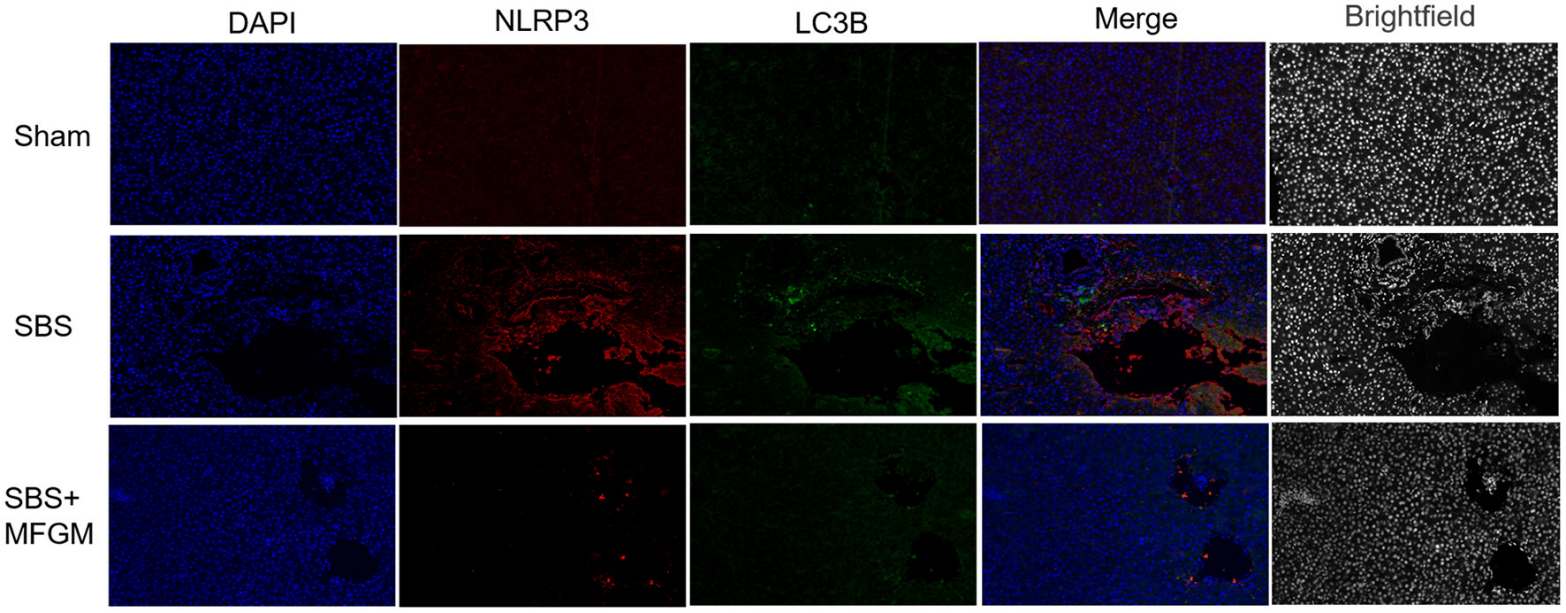
Immunofluorescence staining of NLRP3 and LC3B in the liver. DAPI (blue), NLRP3 (red), LC3B (green), Scale bar: 100 μm. NLRP3, nucleotide-binding oligomerization domain leucine-rich repeat and pyrin domain-containing protein 3; SBS, short bowel syndrome; LC3, microtubule-associated protein1 light chain 3; MFGM, milk fat globule membrane; SBS, short bowel syndrome.

The immunofluorescence staining showed that the fluorescence intensity of NLRP3 and LC3B in the SBS group was obviously enhanced than the other two groups (Figure 5). Also, NLRP3 and LC3B were closely distributed in the same area. The expression of autophagy proteins, Beclin-1 and LC3B, were positively correlated with the NLRP3 levels, respectively (Supplementary Figure S2).

## Discussion

In this study, we found that rats developed significant liver injuries including hepatocyte ballooning, steatosis, and inflammation after massive small bowel resection. Enteral supplementation with MFGM alleviated SBS-associated liver injury, reflected by the lower levels of hepatic steatosis, inflammatory infiltration and ballooning, which might be related to the reduced endotoxin translocation to a certain extent and the inhibition of the activation of both autophagy and NLRP3 inflammasome pathways.

To date, this is the first study to explore the changes of the NLRP3 inflammasome pathway in the liver of the SBS rats. The significantly higher expression of NLRP3, ASC, and Caspase-1 in the SBS group demonstrated NLRP3 inflammasome activation in the setting of SBS-ALD. We also found that the expression of IL-1β was positively correlated with the liver ballooning scores and neutrophil infiltration, as well as increased MPO activity. Supplementation of MFGM to SBS rats presented with lower levels of liver injuries as well as the reduced expressions of the NLRP3 inflammasome pathway proteins. Various studies have suggested that NLRP3 might be regulated by MFGM or some of its components, such as sphingolipids and MUC1 (22, 23). Ng et al. found that the NLRP3 expression was significantly increased in the stomachs of *muc1*^−/−^ but not wild-type mice infected with *H. pylori*, and found that MUC1 inhibited NLRP3 inflammasome activation by negative regulation of toll-like receptor signaling (24). Moreover, Li et al. found that MFG-E8 had a protective role in relieving lipid accumulation and inflammatory response through binding to apoptosis signal-regulating kinase 1 (ASK1) and inhibiting its dimerization and phosphorylation in the pathogenesis of the non-alcoholic fatty liver disease (25). As the downstream inflammatory cytokine of the NLRP3 pathway, IL-1β expression was found to be lower in SBS + MFGM rats than SBS group. A former study showed that IL-1β induced the recruitment of neutrophils to produce oxygen free radicals and proteolytic enzymes through widely expressed interleukin-1 receptor type1(IL-1R1) in the liver leading to the development of inflammation (26). Consistent with this study, we found that the decreased level of IL-1β was accompanied by the ameliorated steatosis and ballooning liver injury and less MPO positive cells when supplementation of MFGM in rats. Additionally, as a complex compound of various bioactive nutrients, supplementation with MFGM strengthened the intestinal barrier function and reduced bacteria translocation rate in SBS rats (18), which contributed to the lower substrate concentration for NLRP3 activation as demonstrated by the lower levels of endotoxin in the liver tissues in SBS + MFGM rats. Hence, to a certain extent, the protective effects of MFGM on SBS-ALD might be mediated by the inhibition of NLRP3 inflammasome activation through various constituents, such as MUC1 and sphingolipids.

Autophagy is a cellular survival mechanism, which plays a vital role in the process of liver injury (27). In this study, we found higher expression of Beclin-1 and LC3B in the SBS group, indicating elevated levels of autophagy in rats at day 15 after massive small bowel resection. Notably, animals supplemented with MFGM showed lower levels of Beclin-1 and LC3B, than SBS rats. Previous study showed that MFGM supplementation improved the intestinal permeability, and therefore reduced the gut derived endotoxin translocated to the liver (18). Furthermore, lactoferrin and whey proteins, as the bioactive components of MFGM, have been shown to reduce oxidative stress (28). As such, the supplementation with MFGM might contribute to the overall downregulation effects on autophagy in SBS through decreasing the levels of liver endotoxin and oxidative stress. However, given the bioactive properties of MFGM components, the underlying autophagy modulating mechanisms by MFGM may be multifaceted. It has been previously suggested that some components of MFGM, such as MUC1 and CD36, could regulate the LC3B, which is critical for the process of autophagy formation (19, 20). Yin et al. revealed that the MUC1 stimulated AMPK activation and promoted lysosomal turnover of LC3B in micro-environments with low glucose levels, but in different conditions, such as SBR, it may reverse the effect on autophagy (29). Another study found that the reconstruction of CD36 in CD36^−^/^−^ mice reduced the level of LC3B through the adenosine monophosphate-activated protein kinase (AMPK) pathway (20). Hence, MFGM might exert regulating effects on the inhibition of autophagy activity through different mechanisms.

Different pattern of the inter-regulation between NLRP3 inflammasome and autophagy has been reported in various diseases (30). Many studies have shown the inhibited autophagy and activated NLRP3 inflammasome in liver diseases, such as drug induced acute liver injury or ischemic-reperfusion liver injury (31, 32). Elevated expressions of both autophagy and NLRP3 inflammasome pathway in the liver were identified in SBS rats in our experiment. Apart from the different pathogenesis of the diseases, the observation time point selected in this study was 14 days post massive SBR, indicating a chronic and repairing status of the SBS rats, which is also different from the above-mentioned acute liver injuries. We also revealed that the expression of LC3B was positively correlated with NLRP3 expression. The fluorescence staining results showed that the NLRP3 appeared to be adjacent to LC3B and even co-localization of the two proteins in some area. Shi et al. found that the immunostaining of endogenous NLRP3 and LC3 overlapped in THP-1 cells stimulated by LPS and ATP, indicating that NLRP3 inflammasome and autophagy might be activated by the same substrates (33). A previous study suggested that the co-localization of LC3B and IL-1β on the autophagic vacuole surface with the confocal microscopy under the starvation condition in neutrophils after LPS + ATP administration and the cytoplasmic IL-1β was transferred to cavity autophagosome by increased autophagy, which promoted the secretion of IL-1β (34). In the setting of SBS, the enteral bacterial endotoxin (mainly LPS), which translocate into the liver through the impaired gut barrier (35), might induce the activation of both the NLRP3 inflammasome and autophagy pathway (36, 37). Moreover, reactive oxygen species (ROS) has been reported to be increased in the liver tissues of rats after the massive bowel resection (38, 39). Thus, the activation of NLRP3 inflammasome and autophagy in SBS-ALD might possibly attribute to the increase of mutual activators, endotoxin and ROS. These findings also suggested that both the NLRP3 inflammasome and autophagy were closely related in the pathophysiological process of SBS-ALD. The activation of NLRP3 inflammasome followed by autophagy seem logical, because autophagy is a cellular survival mechanism to destroy danger substances, and NLRP3 inflammasome, help respond to a danger by recognition of foreign particles from injured cells. As indicated by previous studies (40), we speculated that the activation of autophagy might be a protection against aberrant activation of NLRP3 inflammasome. However, the exact mechanisms underlying the relationship between NLRP3 inflammasome and autophagy in SBS remained to be investigated.

In summary, the present study showed that the NLRP3 inflammasome activation and the increased activity of autophagy were found in the liver of SBS rats, and the enteral supplementation of MFGM alleviated the liver injury by inhibiting the activity of the autophagy-inflammasome pathway in SBS rats. Hence, these findings provided a mechanistic basis for understanding the effects of the MFGM supplementation on liver injury in SBR-ALD rats and offer new insight into the potential therapeutic strategy of SBS-ALD.

## Data Availability Statement

The original contributions presented in the study are included in the article/Supplementary Materials, further inquiries can be directed to the corresponding author/s.

## Ethics Statement

The animal study was reviewed and approved by the Animal Committee of Xinhua Hospital of Shanghai Jiao Tong University School of Medicine.

## Author Contributions

JW: conceptualization. JW, ZY, SH, YL, HC, and YN: investigation and writing—review and editing. JW, ZY, and SH: writing—original draft preparation. All authors have read and agreed to the published version of the manuscript.

## Funding

This work was supported by Shanghai Pujiang Program (18PJD032) and Shanghai Municipal Health System Important and Weak Discipline (Clinical Nutrition) Project (No. 2019ZB0102).

## Conflict of Interest

The authors declare that the research was conducted in the absence of any commercial or financial relationships that could be construed as a potential conflict of interest.

## Publisher's Note

All claims expressed in this article are solely those of the authors and do not necessarily represent those of their affiliated organizations, or those of the publisher, the editors and the reviewers. Any product that may be evaluated in this article, or claim that may be made by its manufacturer, is not guaranteed or endorsed by the publisher.

## Supplementary Material

Supplementary Figure S1 Correlation analysis between IL-1β expression and hepatic pathology scores.

Supplementary Figure S2 Correlation analysis between the autophagy proteins of Beclin-1 **(A)**, P62 **(B)**, LC3B **(C)**, and NLRP3, respectively.
